# Reduced graphene oxide promoted by SnO_2_ for photodegradation of tetracycline in water

**DOI:** 10.1038/s41598-025-32230-4

**Published:** 2025-12-24

**Authors:** Asrin Bahrami, Donya Mohammadi, Faranak Akhlaghian

**Affiliations:** Department of Chemical Engineering, Faculty of Engineering, University of Kurdistan, Sanandaj, Iran

**Keywords:** Photocatalyst, Reduced graphene oxide, Tetracycline, Tin oxide, Visible light, Chemistry, Environmental sciences, Materials science, Nanoscience and technology

## Abstract

Reduced graphene oxide, with a high specific surface area and conductivity, also exhibits photocatalytic activity, and its photocatalytic performance improves with the addition of semiconductors. In this study, SnO_2_/reduced graphene oxide (RGO) composite was applied for tetracycline removal from water under visible light irradiation. The photocatalytic performance of RGO was enhanced through SnO_2_ deposition. RGO was synthesized using Hummer’s method and subsequently impregnated with SnCl_2_ solution to deposit SnO_2_. The effect of SnO_2_ loading was investigated, and the calcination temperature was set at 400 °C. FTIR, XRD, Raman spectroscopy, SEM, TEM, BET, PL, and DRS analyses characterized the SnO_2_/RGO photocatalyst. SEM and TEM confirmed RGO layered morphology. PL analysis revealed an electron-hole recombination peak at 394 nm. DRS results showed a reduction in the band gap of SnO_2_/RGO composite compared to pristine SnO_2_ and RGO. The effects of photocatalyst dosage, initial tetracycline concentration, and pH were studied and optimized using Design-Expert software (version 11) to maximize degradation efficiency. Under optimized conditions, with a tetracycline concentration of 10 mg/L, catalyst dosage of 4 g/L, pH of 7, and 10 min exposure to a 125 W LED lamp, 92% degradation was achieved. Kinetic analysis followed a second-order rate model. The reusability and regenerability of the SnO_2_/RGO composite were evaluated over five cycles, yielding tetracycline removal efficiencies of 91%, 73.25%, 65.7%, 60.3%, and 57%, respectively. Tetracycline removal was further assessed in the presence of Na^+^, Cl^−^, Mg^2+^, and NO_3_^−^, as well as using tap water. Compared with previous studies, this system demonstrated comparable removal efficiency within a substantially shorter reaction time under visible light irradiation, underscoring its economic potential for water treatment applications.

**Supplementary Information:**

The online version contains supplementary material available at 10.1038/s41598-025-32230-4.

## Introduction

Access to clean and safe water is essential for public health and sustainable development. Rapid industrialization and population growth have introduced numerous synthetic compounds into the environment, many of which enter aquatic systems through wastewater discharge. Major contributors to water contamination include agricultural activities and industrial sectors such as oil and petrochemical, pharmaceutical, pulp and paper, and food processing industries^1–4^.

The global consumption of antibiotics has risen markedly due to their essential role in preventing and treating microbial infections. Because these compounds are poorly absorbed in humans and animals, a substantial portion is excreted and released into the environment, where they persist in aquatic systems and disrupt ecological balance. Tetracycline (TC), one of the most commonly used broad-spectrum antibiotics in both human and veterinary medicine, interferes with photosynthesis in aquatic plants and bioaccumulates in aquatic organisms, facilitating its transfer through the food chain. Even at low concentrations, TC in water can promote the development of antibiotic-resistant bacteria. In humans, TC exposure has been linked to gastrointestinal disturbances, reduced appetite, nausea, vomiting, diarrhea, tooth discoloration in children, and adverse effects on fetal bone development^4–9^.

Conventional methods for tetracycline removal from wastewater include precipitation, activated sludge, adsorption, biological treatment, membrane filtration, and advanced oxidation processes (AOPs) such as Fenton, photocatalysis, ozonation, and UV/H_2_O_2_^10,11^. Although membrane filtration can achieve complete removal, it is costly and susceptible to membrane fouling (Table 1). Adsorption methods suffer from adsorbent saturation and the need for regeneration. Biological treatment is often slow and less reliable (Table 1)^10–18^.

**Table 1:** Tetracycline removal methods: advantages and disadvantages^10–18^.

TC wastewater removal method	Advantages	Disadvantages
Precipitation and Activated Sludge	Already available infrastructure and low additional cost	Low efficiency, Transfer to land, Promotes antibiotic gene spreading
Adsorption	High efficiency, Simple, Easy to scale up, non-toxic by-products	Adsorbent saturation and regeneration, Competitive adsorption of other materials, High cost of advanced adsorbents
Advanced Oxidation Processes	Degrade TC into smaller molecules or mineralize completely, suitable for low concentration, Fast removal	High energy demand, Chemical costs, Possible formation of toxic by-products, Required controlled pH and operating conditions
Membrane filtration	High removal efficiency, Suitable for a large spectrum of pollutants,	Expensive capital and operating costs, Membrane fouling, Treatment of the produced concentrated brine
Biological	Eco-friendly and sustainable, Degradation not separation, Low energy demand	Slow and inconsistent, Possible of incomplete degradation, Risk of maintaining or spreading resistance genes

Photocatalysis, a widely studied AOP, involves irradiating a photocatalyst with light to transfer electrons from the valence band to the conduction band, generating e^−^/h^+^ pairs. When the absorbed photon energy exceeds the semiconductor band gap, these charge carriers are produced; however, rapid e^−^/h^+^ recombination dissipates energy as heat, limiting the degradation efficiency. Effective photocatalysis requires separation of e^−^/h^+^ pairs, enabling their interaction with water and dissolved oxygen to form reactive species (e.g., ·OH, O_2_^−^·, HOO·), which degrade pharmaceutical pollutants^19–21^.

Numerous studies have demonstrated high-efficiency photocatalytic degradation of tetracycline in aqueous media. Table 2 summarizes some of these findings^14,15,22–30^.

**Table 2:** Reviews of works for the photocatalytic removal of tetracycline.

No.	References	Year	Photocatalyst
1	Yaun et al.^22^	2021	Phosphated-TiO_2_
2	Sharma et al.^23^	2022	Cu_2_O coupled with TiO_2_ nanotubes
3	Oluwole and Olatunji^24^	2022	3 wt% SnO_2_/g-C_3_N_4_
4	Ghosh et al.^25^	2023	RGO-CdTe
5	Gogoi and Chowdhury^26^	2023	ZnTiO_3_
6	Samy et al.^27^	2023	Olitorius derived biochar/Bi_12_O_17_Cl_2_
7	Liu et al.^14^	2024	TiO_2_/p-biochar
8	Rabeie and Mahmoodi^28^	2025	COF/ZIF(ZnFe)/CoFe_2_O_4_
9	Chen et al.^29^	2025	Gd_2_O_3_/Bi_2_W_x_Mo_1 − x_O_6_
10	Brik et al.^30^	2025	SiNW/CeO_2_/NiO
11	Mandanipour et al.^15^	2025	MIL-53(Fe)-(COOH)@Fe₂O₃

Graphene, discovered in 2004, has attracted considerable attention due to its exceptional mechanical, electrical, thermal, and optical properties, along with its two-dimensional structure composed of sp^2^ hybridized carbon atoms. Each carbon atom forms three sigma bonds in a single atom-thick honeycomb lattice. Graphene’s common synthesis methods include chemical vapor deposition, mechanical exfoliation, liquid phase exfoliation, and pyrolysis^31–35^. In the Hummer’s method, a type of liquid phase exfoliation, graphite oxide is first exfoliated into graphene oxide, which is subsequently reduced to graphene^36^. With its high surface area and low band gap, graphene facilitates charge transfer and visible light absorption, suppresses e^−^/h^+^ recombination, and enhances photocatalytic performance^31–35^. The photocatalytic activity of graphene is also enhanced by coupling it with semiconductor materials, which facilitate charge separation through a photosensitization mechanism^37^. Tin (IV) oxide (SnO_2_), a semiconductor with a band gap of 3.6 eV, has been widely studied for photocatalytic applications^38^. Several previous studies incorporated graphene onto a semiconductor structure, such as SnO_2,_ to degrade organic contaminants^39,40^. In contrast, the present study investigates the deposition of SnO_2_ on reduced graphene oxide (RGO) support for the photocatalytic removal of tetracycline from water. Deposition of SnO_2_ markedly enhanced the photocatalytic activity of RGO, leading to improved tetracycline efficiency. The SnO_2_/RGO composite was characterized using FTIR, SEM, XRD, TEM, BET, PL, and DRS techniques. Its removal efficiency was evaluated under various operating conditions to identify the optimum parameters for maximum tetracycline removal.

## Materials and methods

### Materials

TC was obtained from Sigma-Aldrich. Graphite powder, nitric acid (HNO_3_), potassium permanganate (KMnO_4_), sulfuric acid (H_2_SO_4_), ascorbic acid (C_6_H_8_O_6_), hydrogen peroxide (H_2_O_2_), ethanol (C_2_H_5_OH), hydrochloric acid (HCl), ammonia solution (NH_4_OH), and tin (II) chloride (SnCl_2_) were purchased from Merck and used.

### SnO_2_/RGO photocatalyst synthesis

#### RGO synthesis

RGO was synthesized by Hummer’s method. Graphite powder (1 g) was added to a mixture of 18 mL HNO_3_ and 46 mL H_2_SO_4_, and the solution was stirred in an ice bath for 45 min. KMnO_4_ (5 g) was gradually added while keeping the temperature below 20 °C, followed by stirring for 90 min. The reaction continued at 35 °C for 15 min. Subsequently, 100 mL of deionized water was added, the temperature was increased to 98 °C, and stirring was continued for 15 min. After 1 h, the suspension turned dark brown. Then, 120 mL of deionized water and 15 mL of H_2_O_2_ (30 wt%) were added simultaneously, forming a yellow dispersion. The resulting precipitate was centrifuged and washed with 100 mL of dilute HCl (1:100 v/v) to remove residual metal ions, followed by rinsing with deionized water until the supernatant pH reached 5–6. The final product (graphene oxide, GO) was dried in an oven at 70 °C for 12 h^36^.

To obtain RGO, the dried intermediate (graphene oxide, GO) was ultrasonically dispersed in 500 mL of deionized water. Ascorbic acid (5 g) was added, and the pH was adjusted to 10 using NH_4_OH. The suspension was stirred at 95 °C for 2 h, then centrifuged to recover the precipitate. After sequential washing with deionized water to neutral pH and ethanol, the precipitate was dried at 70 °C for 12 h^36,41^.

#### SnO_2_/RGO synthesis

Tin chloride (0.05 g) was dissolved in 100 mL of deionized water, after which 0.1 g of RGO powder was added to the solution. The mixture was sonicated for 20 min, centrifuged, and the precipitate was dried at 100 °C for 12 h. The dried material was then calcinated at 400 °C for 2 h to yield the SnO_2_/RGO photocatalyst.

### Characterization instrument

Fourier transform infrared spectroscopy (FTIR) was conducted using a Vector 22 spectrometer (Bruker) to identify and analyze surface functional groups. X-ray diffraction (XRD) was employed to examine the crystalline structure of the photocatalyst. Diffraction patterns were recorded over a 2θ range of 10 to 80° with a step size of 0.02°, using a Cu kα anode (λ = 0.154 nm, 40 kV, 40 mA) on an X’Pert MPD diffractometer (Philips). The structural features of the synthesized graphene were assessed using Raman spectroscopy (Unidorn). Elemental analysis was performed by inductively coupled plasma optical emission spectroscopy (ICP-OES) using a 730-ES instrument (Varian). Surface morphology was visualized using scanning electron microscopy (SEM, MIRA3, TESCAN). Transmission electron microscopy (TEM, Zeiss-EM) images were captured at an electron voltage of 100 kV. The photoluminescence (PL) spectrum was recorded using Varian spectrometer to evaluate e^−^/h^+^ recombination rates. Diffuse reflectance spectroscopy (DRS) was carried out using an AvaSpec-2048TE spectrometer (Avantes).

### Photocatalyst tests

The experimental setup has been described in detail in the previous study^42^. Photocatalytic degradation tests were carried out under visible light irradiation using a 125 W LED lamp. In a typical experiment, the SnO_2_/RGO photocatalyst (4 g/L) was added to 50 mL of TC solution with an initial concentration of 5 mg/L. The suspension was stirred for 10 min, followed by centrifugation to separate the photocatalyst. The remaining TC concentration was determined using a TG 80 + spectrophotometer (PG Instruments) based on the Beer-Lambert law. The maximum absorption wavelength (λ_max_) for TC was 357 nm. The removal efficiency was calculated using Eq. (1)^42,43^:

1 $${\text{TC removal}}\left( {\%} \right)=\frac{{{{\mathrm{C}}_0} - {\mathrm{C}}}}{{{{\mathrm{C}}_0}}} \times 100$$

where C_0_ is the initial TC concentration (mg/L), and C is the concentration after photocatalytic treatment.

### Experimental design

Experimental design involves systematic planning and execution of tests to evaluate the effects of multiple independent variables on a response. Once a reliable model is established, it can be used to predict responses within defined parameter ranges^44,45^. In this study, Design Expert software (version 11) was used. Response surface methodology (RSM) based on a Box-Behnken design was employed to model and optimize the TC degradation process. The independent variables included TC concentration (5–55 mg/L), photocatalyst dosage (0.2–4 g/L), and pH (2–10). Reaction time (10 min) and light intensity (125 W LED lamp) were kept constant. The response was TC removal efficiency. Table 3 summarizes the experimental conditions and corresponding responses.

**Table 3:** Experimental design and responses.

No	Input variable			TC removal (%)	
	Photocatalyst dose (g/L)	pH	TC initial concentration (mg/L)	Experimental	Modeling
1	2.1	7	30	70.50	68.61
2	2.1	7	30	70.40	68.61
3	4	7	55	65.98	66.17
4	2.1	7	30	70.00	68.61
5	0.2	10	30	6.71	6.92
6	0.2	7	55	24.60	24.75
7	4	7	5	92.00	92.18
8	4	10	30	37.68	37.68
9	0.2	2	30	9.63	9.54
10	2.1	10	5	36.26	37.00
11	4	2	30	31.84	31.84
12	2.1	7	30	67.33	68.61
13	2.1	7	30	67.00	68.61
14	0.2	7	5	33.27	31.4
15	2.1	10	55	28.98	28.24
16	2.1	2	55	58.98	56.53
17	2.1	7	55	59.34	62.36
18	0.2	10	55	1.92	1.71
19	2.1	7	5	72.00	71.12
20	2.1	2	30	60.38	62.79
21	0.2	2	5	10.89	10.98
22	0.2	7	5	29.68	31.4
23	4	7	30	81.43	81.05

## Results and discussions

### Optimization of SnO_2_/RGO

**Fig. 1:**
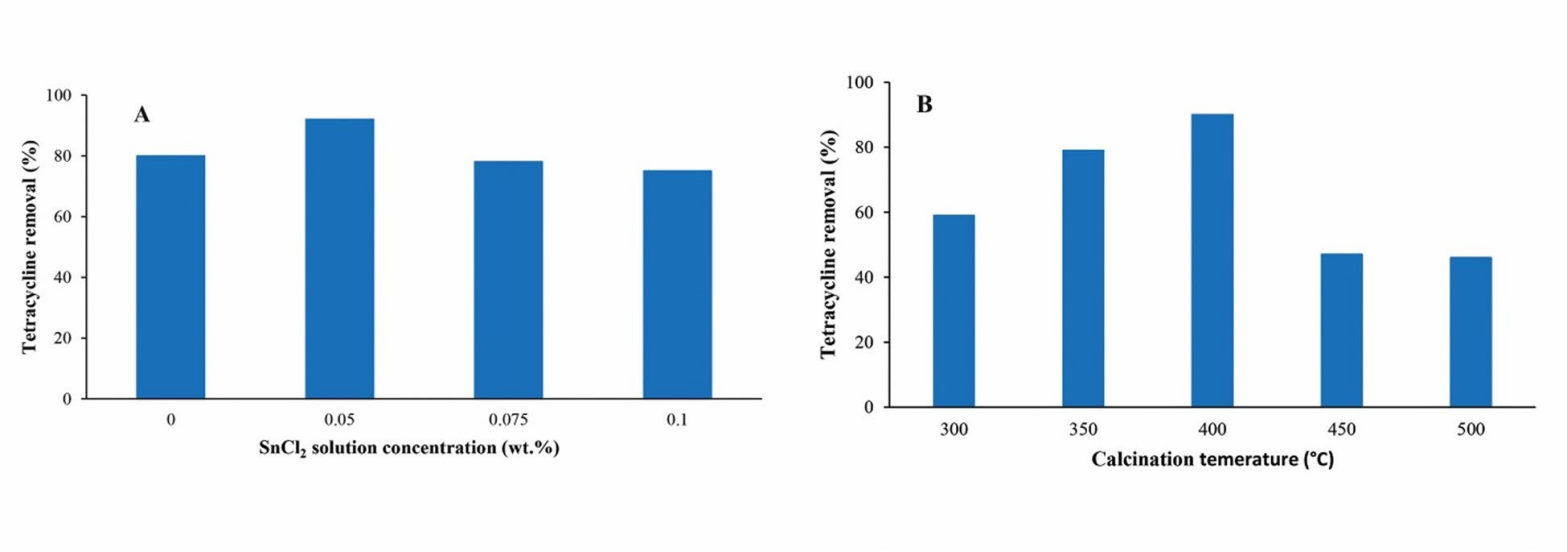
Effects of (**A**) SnCl_2_ concentration and (**B**) photocatalyst calcination temperature on TC removal; Operating conditions: initial TC concentration 10 mg/L, pH 7, catalysts dosage 2.1 g/L, reaction time 10 min; in (**A**) calcination temperature 400 °C; (**B**) SnCl_2_ concentration 0.05 wt%.

During the synthesis of the photocatalyst, SnCl_2_ solutions with concentrations of 0, 0.05, 0.07, and 1 wt% were tested. As shown in Fig. 1A, the 0.05 wt% SnCl_2_ concentration resulted in the highest TC removal. Increasing the SnCl_2_ content from 0 to 0.05 wt% enhanced TC degradation, likely due to the increased number of tin active sites. However, concentrations above 0.05 wt% led to interactions between adjacent active sites, reducing TC adsorption and consequently diminishing photocatalytic efficiency.

Following the optimization of tin content, the effect of calcination temperature was evaluated at 300, 350, 400, 450, and 500 °C. As depicted in Fig. 1B, the highest TC removal was observed at 400 °C. Based on these findings, all subsequent SnO_2_/RGO syntheses were conducted using 0.05 wt% SnCl_2_ as the precursor and a calcination temperature of 400 °C.

### Characterization of SnO_2_/RGO

**Fig. 2:**
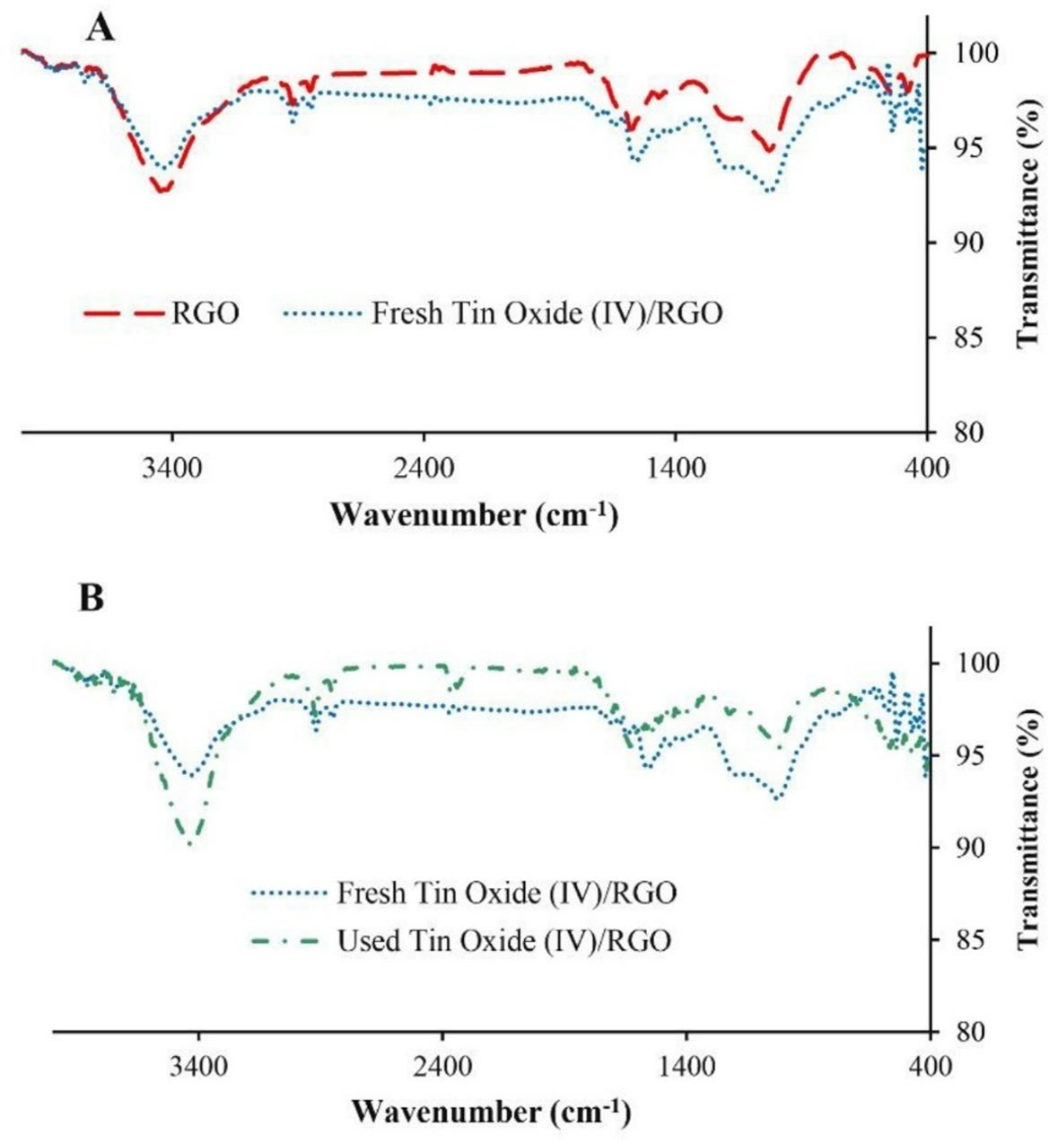
FTIR spectra of (**A**) RGO and fresh SnO_2_/RGO; (**B**) fresh and used SnO_2_/RGO.

The weight content in the composite (SnO_2_/RGO), determined by ICP-OES, and was 4.43%. FTIR, XRD, and Raman spectroscopy were done to identify the functional groups and structure of the SnO_2_/RGO. In the FTIR spectrum of RGO (Fig. 2A), characteristic absorption bands appeared at 3446 cm^−1^ (O–H stretching), 2921.16 cm^−1^ (C–H stretching), 1578.84 cm^−1^ (C = C stretching), and 1027.53 cm^−1^ (C–O stretching). In the spectrum of SnO_2_/RGO, these bands exhibited higher intensities, attributed to the presence of Sn species. The bands at 573.78, 537.17, and 474.80 cm^−1^ were assigned to Sn-O bonds^46–48^. The FTIR spectrum of SnO_2_/RGO after reaction (Fig. 2B) showed absorption bands at 3445.64 cm^−1^ (O–H stretching), 2920.96 and 2851.59 cm^−1^ (C–H stretching), 1624.48 cm^− 1^ (C=C stretching), 1024.76 cm^−1^ (C–O stretching), and 548.21, 475.10, and 415.90 cm^−1^, corresponding to Sn–O vibrations. The decreased intensities relative to the fresh sample can be related to the tetracycline adsorption.

**Fig. 3:**
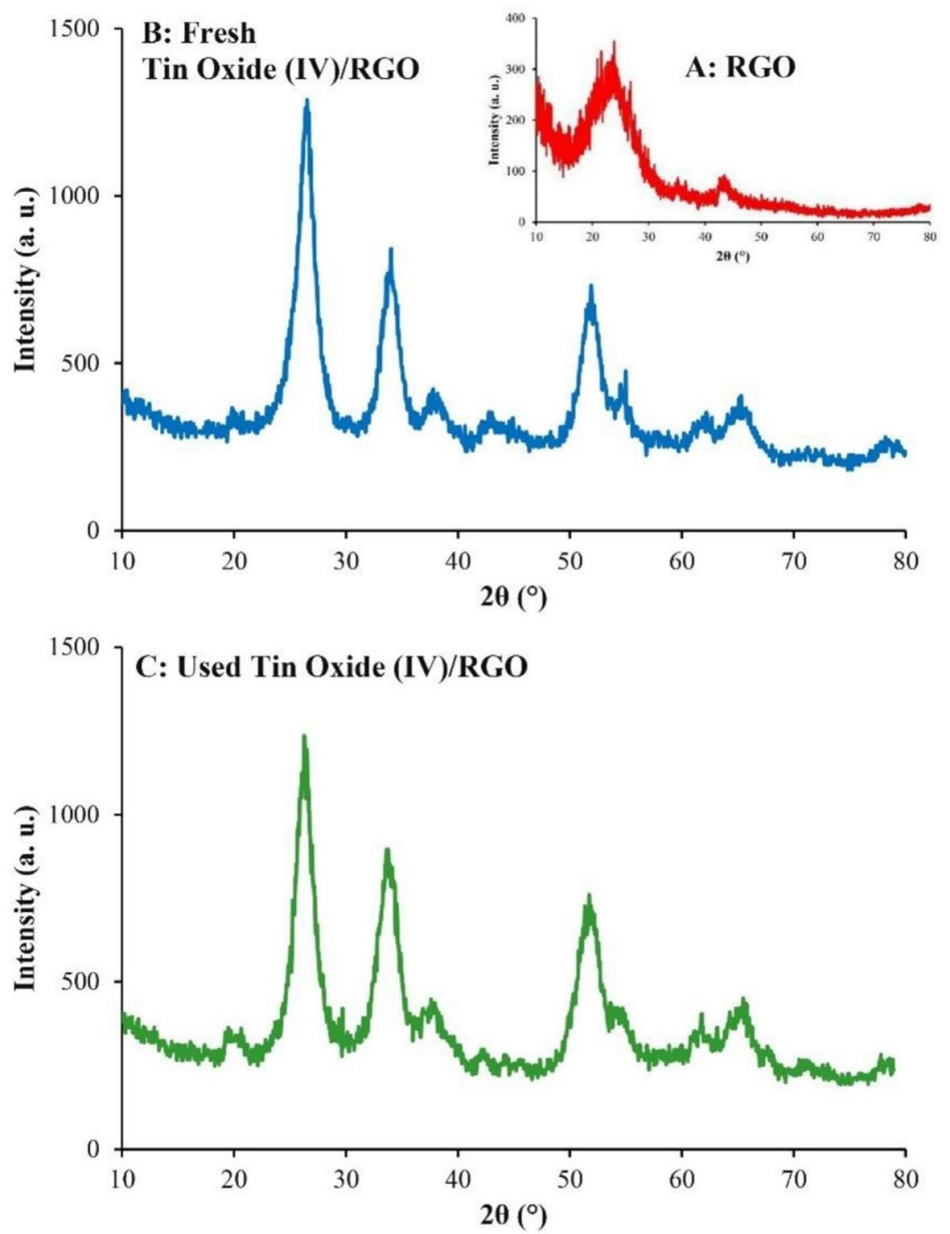
XRD pattern of (**A**) RGO; (**B**) fresh SnO_2_/RGO; and (**C**) used SnO_2_/RGO.

The XRD pattern of RGO (Fig. 3A) displayed peaks at 11.85°, 25°, and 42.85°, which is consistent with the literature^46^. In the XRD pattern of fresh SnO_2_/RGO (Fig. 3B), peaks at 26.39°, 34.04°, and 51.89° corresponded to the tetragonal SnO_2_ phase (JCPDS File No. 72-1147)^49^. RGO peaks were absent, likely due to the dominance of SnO_2_ crystallinity and strong diffraction intensity. Crystallite size was calculated using the Scherrer equation: $\:\mathrm{L}=\frac{\mathrm{k}{\uplambda\:}}{{\upbeta\:}\mathrm{c}\mathrm{o}\mathrm{s}\left({\uptheta\:}\right)}$, where λ is the X-ray wavelength (nm), β is the full width at half maximum in radians, k is the shape factor (typically 0.9), and θ is the diffraction angle^50^. The crystalline size of SnO_2_ in the fresh composite was 10.82 nm. The XRD pattern of the used SnO_2_/RGO (Fig. 3C) showed peaks at 26.29°, 33.79°, and 51.74°, indicative of tetragonal SnO_2_ (JCPDS File No. 72-1147). The intensities of these peaks remained unchanged relative to the fresh sample, confirming no Sn leaching. The crystalline size of SnO_2_ in the used photocatalyst was 12.37 nm, showing a negligible structural change of SnO_2_.

**Fig. 4:**
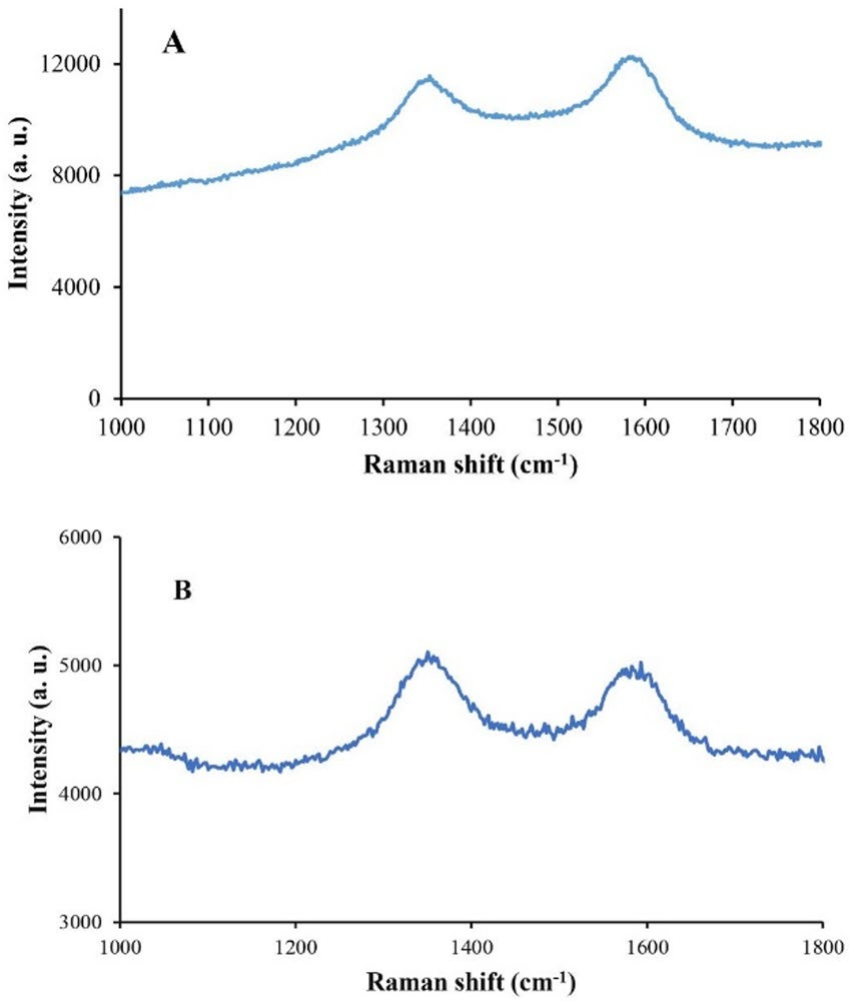
Raman spectra of (**A**) RGO and (**B**) SnO_2_/RGO.

Raman spectra of RGO and SnO_2_/RGO are presented in Fig. 4. The spectrum of RGO (Fig. 4A) featured a D band at 1352.17 cm^−1^, associated with defects, and a G band at 1599.58 cm^−1^, attributed to in-plane vibrations of sp^2^ hybridized carbon atoms. For SnO_2_/RGO (Fig. 4B), D and G bands were observed at 1350.03 and 1529.97 cm^−1^, respectively, with decreased intensity due to SnO_2_ loading and partial reduction of the RGO structure^49^. The I_D_/I_G_ ratios were 0.988 (RGO) and 1.02 (SnO_2_/RGO), indicating increased defect density during SnO_2_ deposition and calcination.

**Fig. 5:**
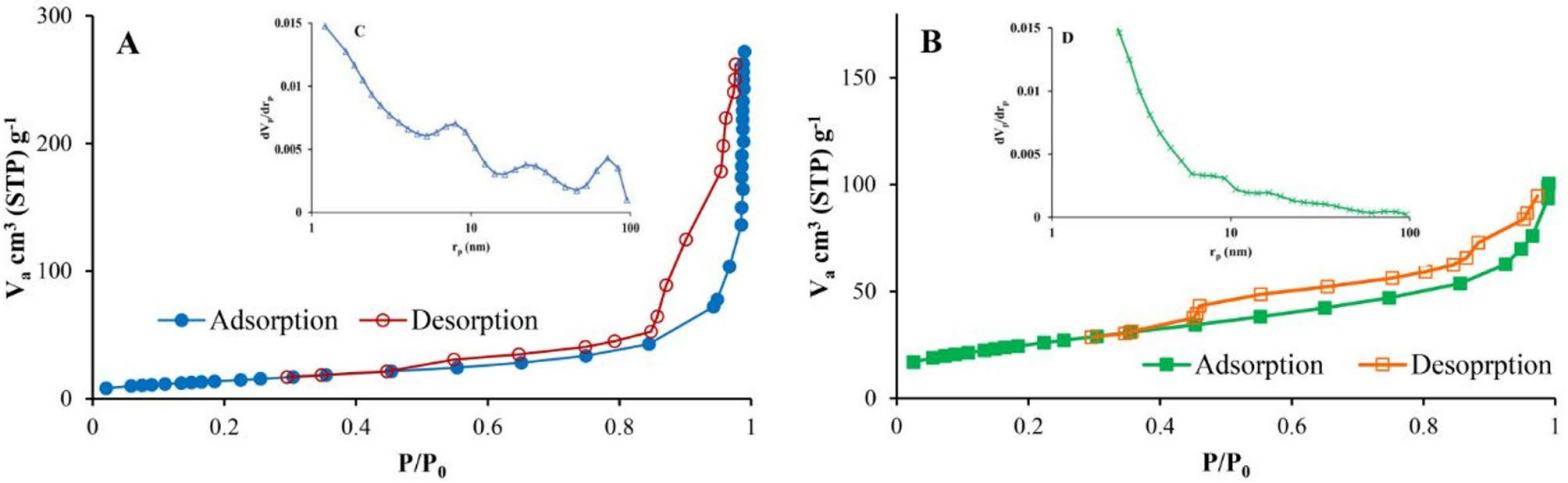
(**A**, **B**) N_2_ adsorption/desorption isotherm for RGO and SnO_2_/RGO; (**C**, **D**) pore size distribution.

Nitrogen adsorption/desorption isotherms (Figs. 5A and B) exhibited type IV behavior with H3 hysteresis loops, indicative of slit-shaped, non-uniform pores^51^. Pore size distribution analysis (Figs. 5C and D) revealed a multimodal distribution for RGO and a unimodal distribution for SnO_2_/RGO, with the most frequent pore diameters at 1.21 nm. Specific surface area, porosity, and average pore diameter values are reported in Table 4.

**Table 4:** Specific surface area, porosity, and average pore diameter of RGO and SnO_2_/RGO.

Sample	Specific surface area (m^2^/g)	Pore volume (cm^3^/g)	Average pore diameter (nm)
RGO	62.68	0.4203	1.21
SnO_2_/RGO	70.493	0.1477	1.21

**Fig. 6:**
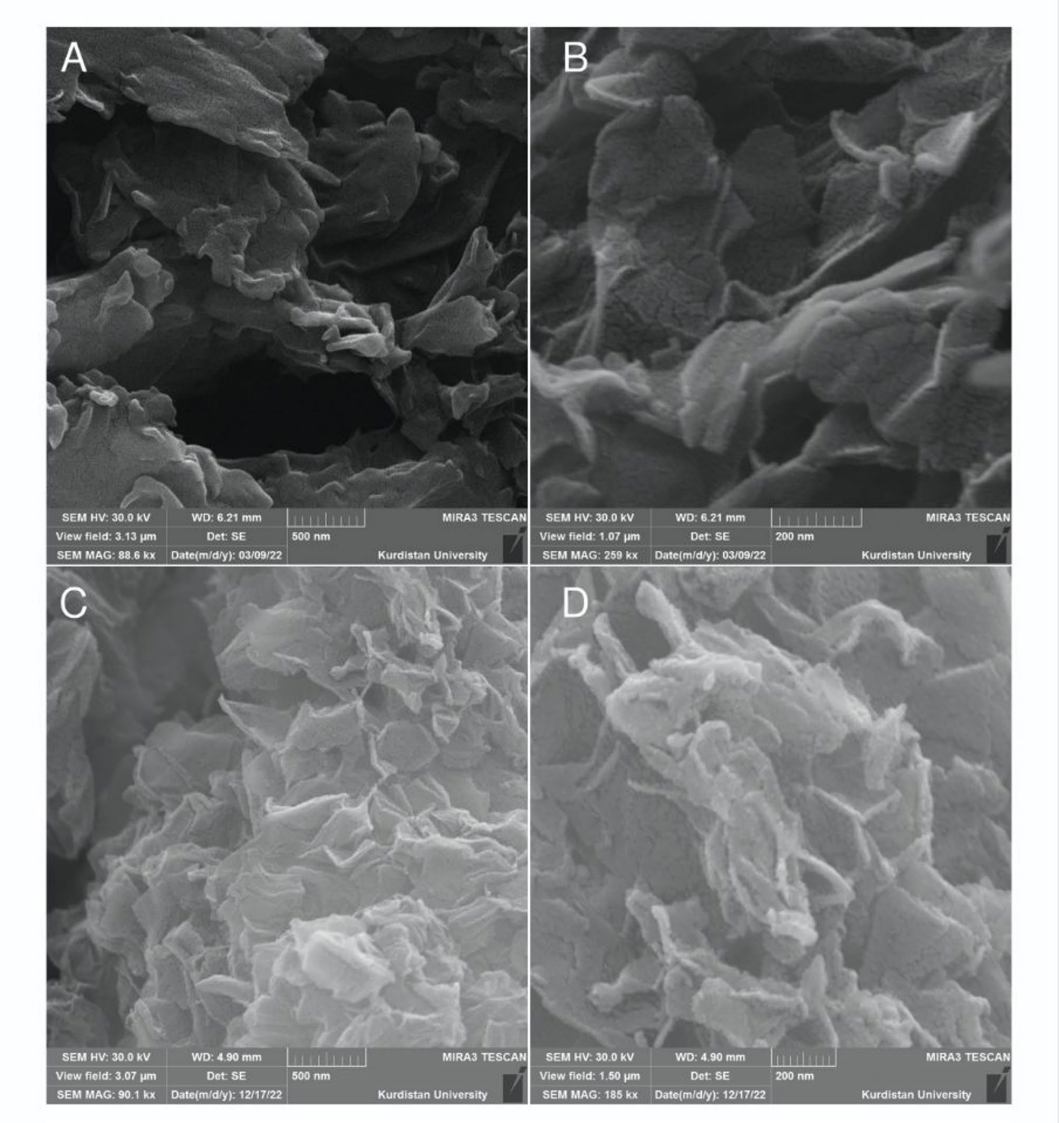
SEM images of (**A**, **B**) RGO and (**C**, **D**) SnO_2_/RGO.

SEM images of RGO with magnifications of 88,500 and 259,000 (Figs. 6A and B) revealed a thin, wrinkled, layered morphology^52^. SEM images of SnO_2_/RGO with magnifications of 90,100 and 185,000 (Figs. 6C and D) displayed bright surface features corresponding to SnO_2_ nanoparticles.

**Fig. 7:**
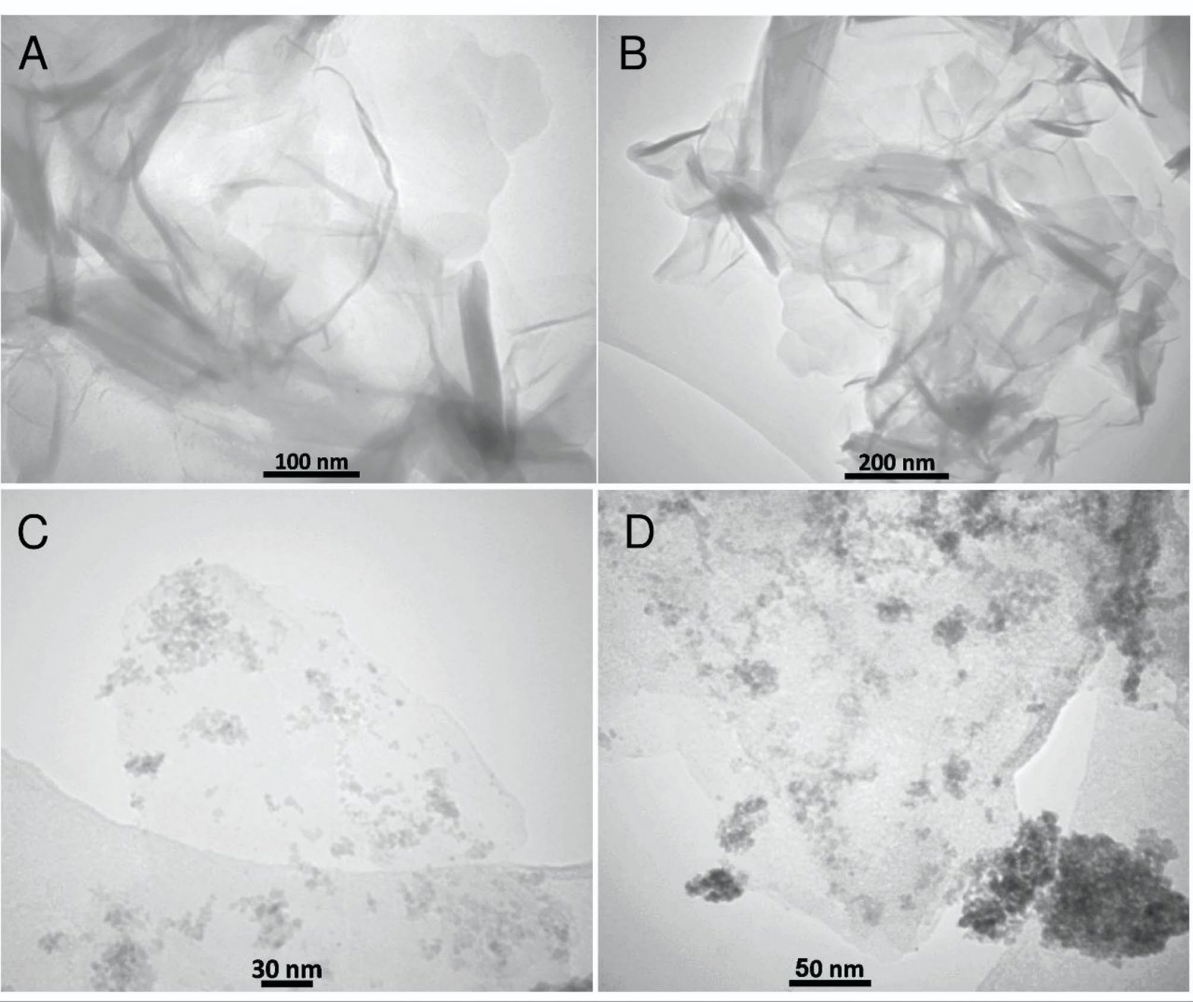
TEM images of (**A**, **B**) RGO and (**C**, **D**) SnO_2_/RGO.

TEM images (Figs. 7A–D) confirmed the layered, transparent morphology of RGO (Fig. 7A, B) and the uniform distribution of SnO_2_ nanoparticles on the graphene surface (Figs. 7C, D) ^49, 52^.

**Fig. 8:**
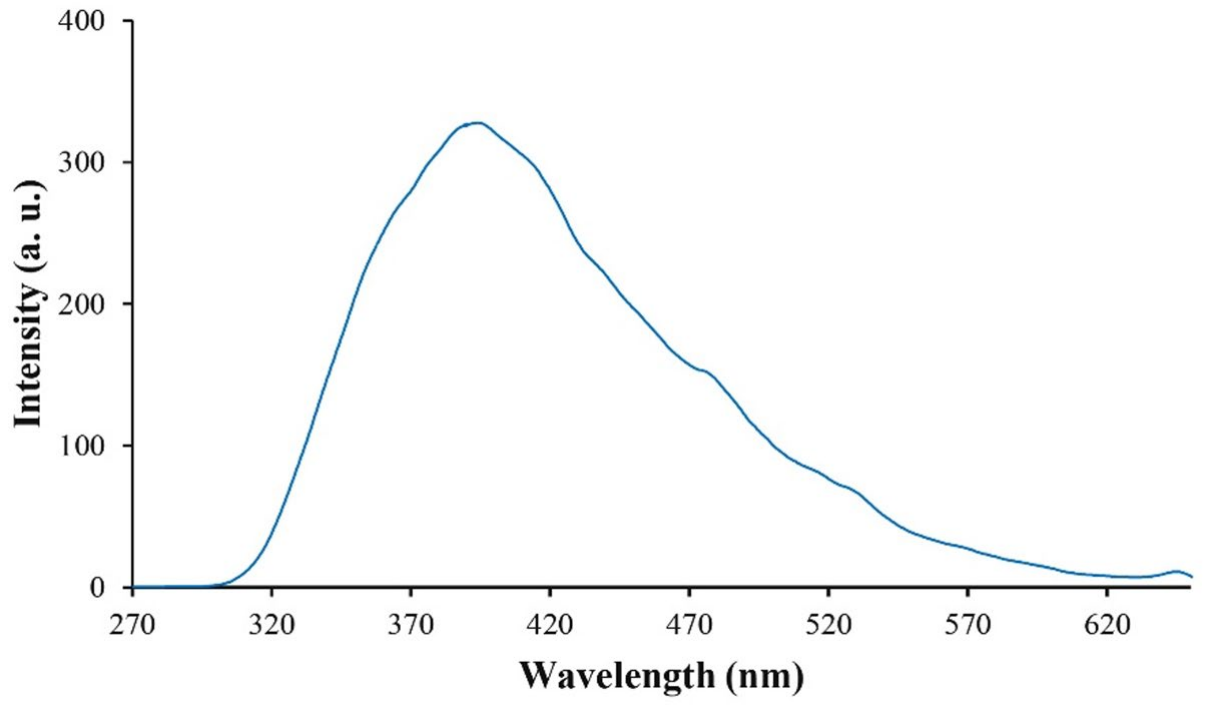
Photoluminescence spectrum of SnO_2_/RGO.

The high electron mobility of RGO suppresses e^−^/h^+^ recombination^53,54^. Photoluminescence spectroscopy of SnO_2_/RGO (Fig. 8), excited at 327.63 nm, exhibited an emission peak at 394 nm, indicating that recombination was maximized. Electron transfer in RGO effectively suppressed the recombination of e^−^/h^+^ and enhanced the photocatalytic activity^54–56^.

**Fig. 9:**
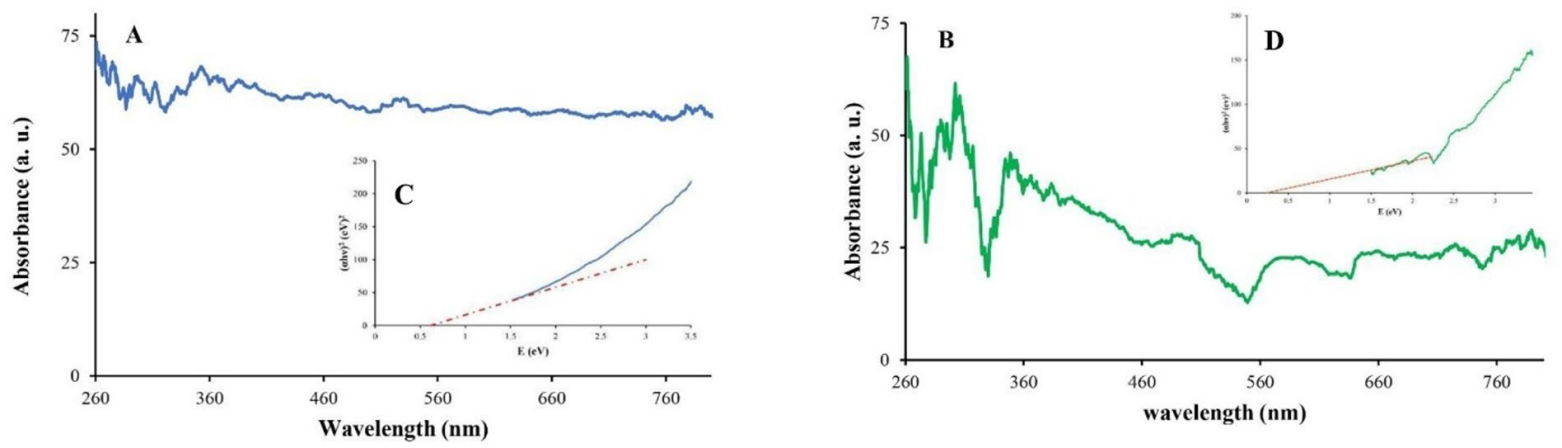
UV–vis DRS spectra of (**A**) RGO, (**B**) SnO_2_/RGO; Tauc plots of (**C**) RGO, (**D**)SnO_2_/RGO.

UV-Vis diffuse reflectance spectra (Figs. 9A and B) confirmed light absorption across both UV and visible regions. Tauc plots (Figs. 9C and D), constructed with photon energy (hν, calculated as 1240/λ) on the x-axis and (αhν)^2^ on the y-axis^48,56–58^, and revealed band gaps of 0.615 eV for RGO and 0.24 eV for SnO_2_/RGO, indicating improved photocatalytic activity of SnO_2_/RGO due to its lower band gap.

### Experimental design

The independent variables in the design were photocatalyst dosage (g/L), pH, and initial TC concentration (mg/L). All experiments were conducted under a fixed irradiation time of 10 min using a 125 W LED lamp. Experimental runs were randomized, and each test was performed in triplicate with averaged values recorded. The investigated ranges were 0.2–4 g/L for catalyst dosage, 2–10 for pH, and 5–55 mg/L for TC initial concentration.

Accurate model selection is critical in experimental design analysis for ensuring predictive reliability. A fourth-order polynomial model was selected, yielding a statistically significant fit with an F-value of 349.92 and p-value < 0.0001 (Table 5)^59,60^. The derived model was Eq. (2):

2 $$\begin{aligned} {\text{TC removal }}\left( \% \right)= & - {\mathrm{3}}0.{\mathrm{97128}}\,+\,{\mathrm{68}}.{\mathrm{49}}0{\mathrm{69A}}\,+\,{\mathrm{18}}.{\mathrm{92293B}}\,+\,0.0{\mathrm{33896C}} \\ \quad & - {\mathrm{8}}.{\mathrm{7}}0{\mathrm{445AB}}\,+\,0.0{\mathrm{74187AC}} - {\mathrm{16}}.{\mathrm{27825}}{{\mathrm{A}}^{\mathrm{2}}} - {\mathrm{1}}.{\mathrm{53981}}{{\mathrm{B}}^{\mathrm{2}}} - 0.00{\mathrm{3}}00{\mathrm{1}}{{\mathrm{C}}^{\mathrm{2}}} \\ \quad & +\,{\mathrm{3}}.{\mathrm{31724}}{{\mathrm{A}}^{\mathrm{2}}}{\mathrm{B}} - 0.0{\mathrm{41937}}{{\mathrm{A}}^{\mathrm{2}}}{\mathrm{C}}\,+\,0.{\mathrm{386157A}}{{\mathrm{B}}^{\mathrm{2}}} - 0.{\mathrm{19}}0{\mathrm{15}}0{{\mathrm{A}}^{\mathrm{2}}}{{\mathrm{B}}^{\mathrm{2}}} \\ \end{aligned}$$

where A, B, and C represent photocatalyst dosage (g/L), pH, and TC initial concentration (mg/L), respectively. The coefficient of determination (R^2^) was 0.9972, and the adjusted R^2^ was 0.9939, indicating a strong agreement between experimental and predicted values. Adequate precision was 63.73, confirming model robustness. The model is valid within the investigated ranges, though extrapolation beyond these boundaries should be approached with caution. Among the studied variables, photocatalyst dosage exhibited the most significant effect on TC removal (F-values A: 692.41, A^2^: 15.20), followed by pH (F-values B: 65.64, B^2^: 267.51), while TC initial concentration showed comparatively the lowest influence (F-values C:20.12, C^2^:2.94).

**Table 5:** The ANOVA of the mathematical model.

Source	F-Value	*p*-value	
Model	298.93	< 0.0001	Significant
A	692.41	< 0.0001	
B	165.64	< 0.0001	
C	20.12	0.0012	
AB	5.17	0.0463	
AC	28.7	0.0003	
A^2^	115.2	< 0.0001	
B^2^	267.51	< 0.0001	
C^2^	2.94	0.1169	
A^2^B	107.83	< 0.0001	
A^2^C	8.09	0.0174	
AB^2^	86.50	< 0.0001	
A^2^B^2^	22.15	0.0008	
Lacko of fit	1.29	0.3920	Not significant

**Fig. 10:**
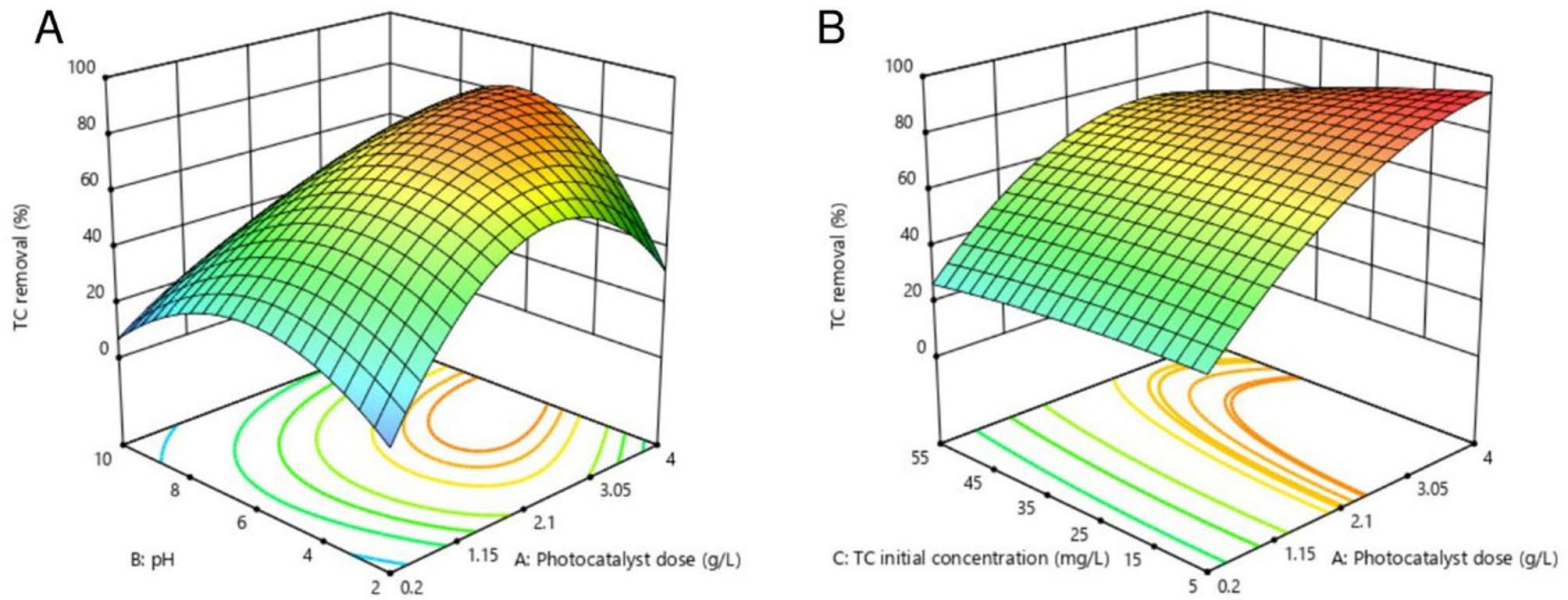
Effects of operating parameters on TC removal: (**A**) interaction between catalyst dosage and pH; (**B**) interaction between catalyst dosage and TC initial concentration.

 Figure 10A depicts the interaction between photocatalyst dosage and pH. At constant pH values, TC removal increased with increasing catalyst dosage, reaching a maximum, after which further increases reduced removal efficiency. This trend was initially attributed to enhanced photogenerated charge carrier formation, which promoted degradation, and later to increased turbidity, which hindered light penetration and facilitated e^−^/h^+^ recombination^61^.

At fixed dosage levels, pH also influenced TC removal efficiency. TC, an amphoteric compound, has dissociation constants (pKa) at 3.2, 7.7, and 9.6. Its species distribution varies with pH: H_3_TC^+^ (cationic) below pH 3.2, H_2_TC (neutral), at 3.2 < pH < 7.7, and HTC^−^ or TC^2−^ (anionic) above pH 7.7. Under acidic conditions, photocatalyst surface protonation induces electrostatic repulsion with cationic TC, limiting adsorption. Similarly, in alkaline environments, repulsion between anionic TC species and negatively charged surfaces reduces removal. Maximum TC degradation was observed near neutral pH (Fig. 10A)^58,61,62^.

 Figure 10B presents the interaction between photocatalyst dosage and initial TC concentration. Increasing TC concentration decreased removal efficiency due to reduced active site availability per molecules. In both cases (Figs. 10A and B), optima for catalyst dosages were observed^62^.

The optimum conditions, selected from the design space, were TC initial concentration 5 mg/L, pH 7, and a photocatalyst dosage of 4 g/L, with a 10 min reaction time. Under these conditions, the experimental and predicted TC removal efficiencies were 92 and 92.18%, respectively, with the model prediction within ± 5% of experimental result.

### Effects of light sources

**Fig. 11:**
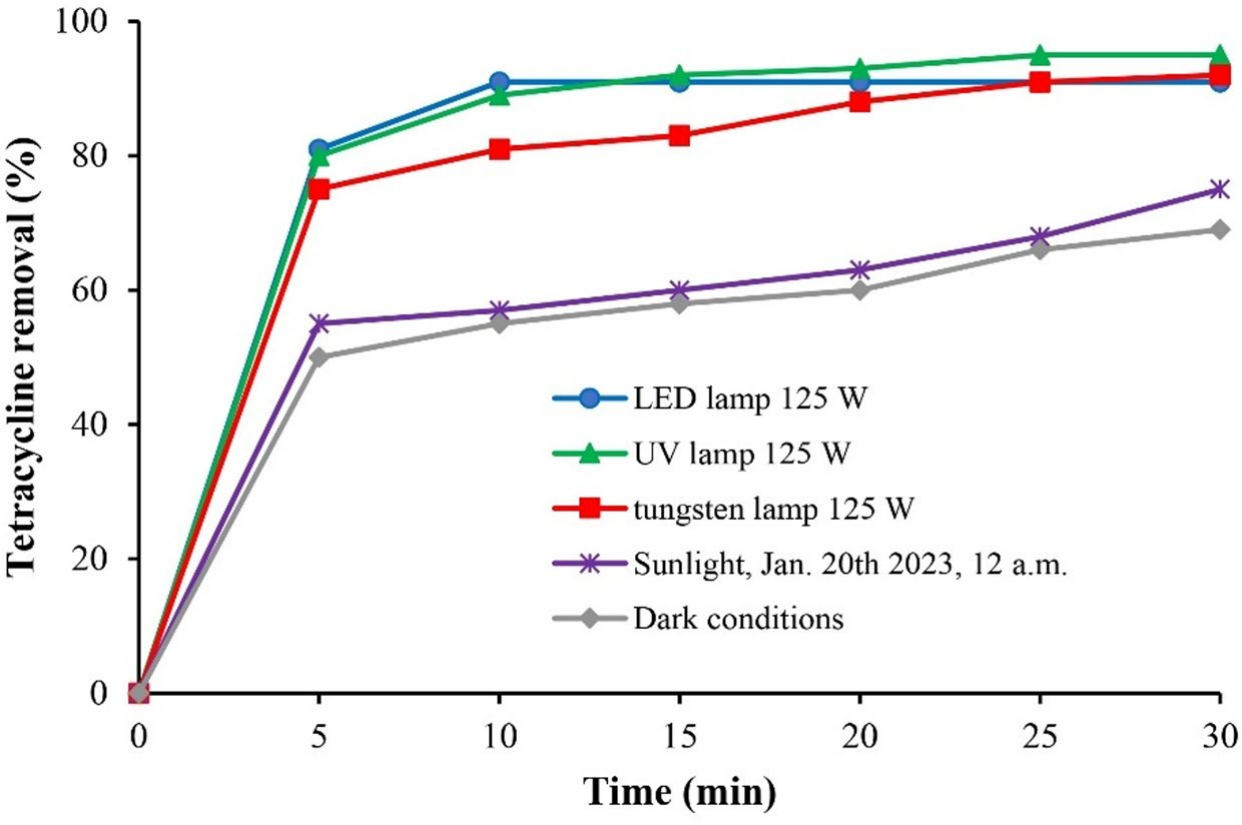
Effects of different light sources on TC removal, operating conditions: TC initial concentration 10 mg/L, catalyst dosage 4 g/L, pH 7, and reaction time 10 min.

Figure 11 illustrates the effect of different light sources on TC photodegradation using the SnO_2_/RGO. Photocatalytic activity was observed under all tested irradiation conditions. Sunlight experiments were performed at 12:00 p.m. on January 20, 2022, in Sanandaj, Iran. Adsorption was the mechanism of TC removal in the dark conditions. The experimental data were fitted to zero, first, and second-order kinetic models. As summarized in Table 6, the photodegradation of TC followed a second-order kinetic model for all light sources. Among the tested light sources, the LED lamp yielded the highest rate constant, indicating superior photocatalytic performance for TC removal. The demonstrated activity of SnO_2_/RGO under artificial and natural visible light suggests promising potential for cost-effective environmental remediation applications.

**Table 6:** Kinetic model fitting for various light sources^42^.

Light source	Model					
	Zero-order model C − C_0_ = k_0_t (3)		First-order model Ln(C/C_0_) = k_1_t (4)		Second-order model 1/C − 1/C_0_ = k_2_t (5)	
	k_0_ (mg/L min)	*R*^2^	k_1_ (min^− 1^)	*R*^2^	k_2_ (L/mg min)	*R*^2^
LED lamp	0.91	0.8313	0.2408	0.9542	0.1011	0.9919
UV lamp	0.89	0.8250	0.2207	0.9346	0.0809	1.0000
Tungsten lamp	0.81	0.8052	0.1661	0.8700	0.04426	0.9478
Sunlight	0.57	0.7763	0.0844	0.7903	0.0133	0.8081
Dark conditions	0.55	0.8176	0.0799	0.8470	0.01229	0.8811

### Reuse and regeneration

**Fig. 12:**
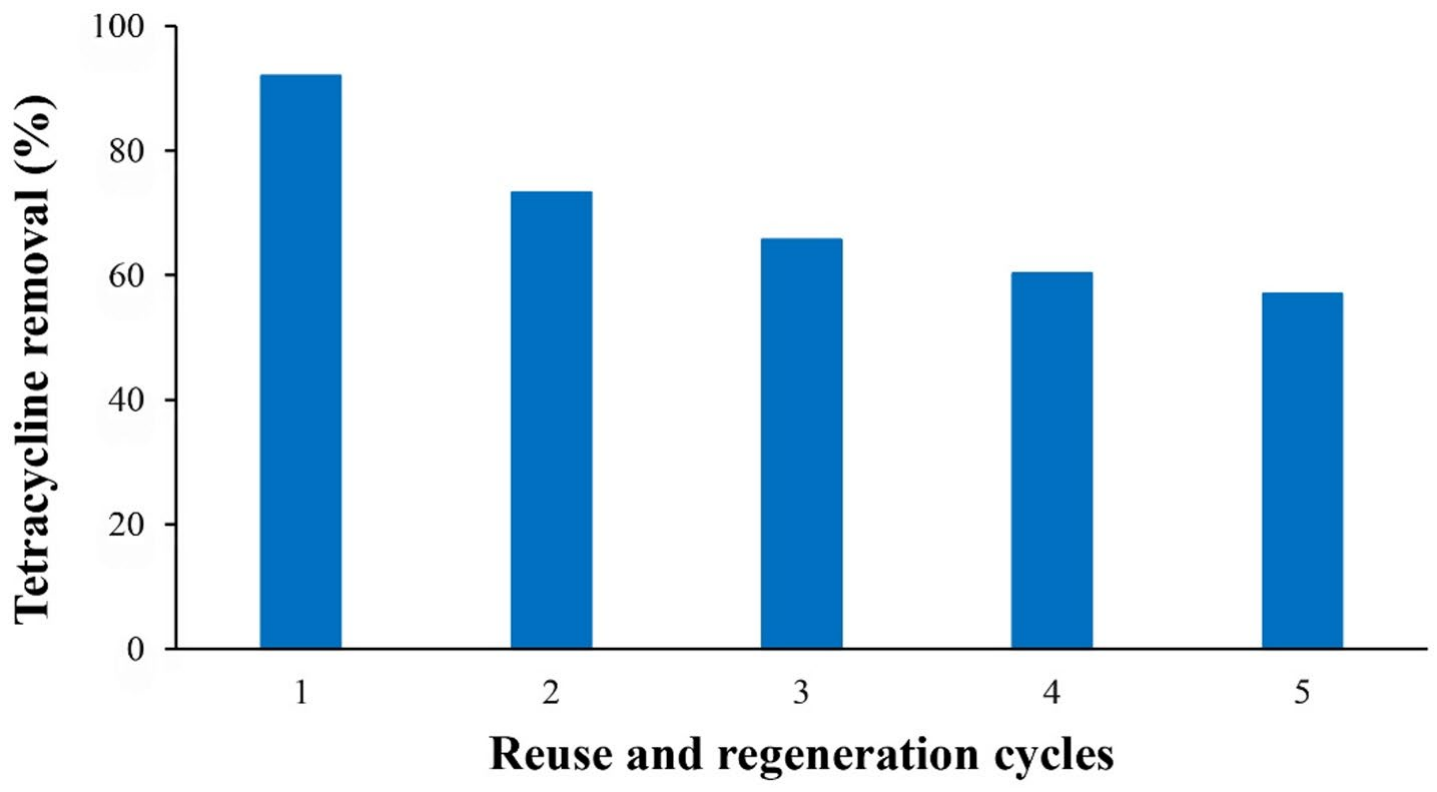
Reuse and regeneration cycles, operating conditions: TC initial concentration 10 mg/L, pH 7, catalyst dosage 4 g/L, time 10 min.

Photocatalyst reusability is a critical factor for practical application. Under these conditions; TC initial concentration 10 mg/L, pH 7, catalyst dosage 4 g/L, reaction time 10 min under irradiation of LED lamp 125 W; the SnO_2_/RGO photocatalyst was recovered by centrifugation, ultrasonically washed in ethanol for 10 min, rinsed with deionized water, and dried. As shown in Fig. 12, TC removal decreased from 91% in the first cycle to 57% in the fifth, indicating partial deactivation of SnO_2_/RGO over repeated use due to TC adsorption according to FTIR results.

### Mineralization and effects of other pollutants

To ensure mineralization of TC during photodegradation reaction, COD and BOD5 analyses were conducted. The photocatalytic reaction was carried out under the following conditions: TC concentration 5 mg/L, pH 7, SnO_2_/RGO dosage 4 g/L, 10 min reaction time, and irradiation by a 125 W LED lamp. COD and BOD5 removal were calculated using Equations. (S1) and (S2) in the Supplementary Material^63,64^. BOD5 and COD removal efficiencies were 100%, and 72.5%; respectively, confirming during photodegradation 72.5% of organic materials were mineralized and converted to CO_2_ and H_2_O.

**Fig. 13:**
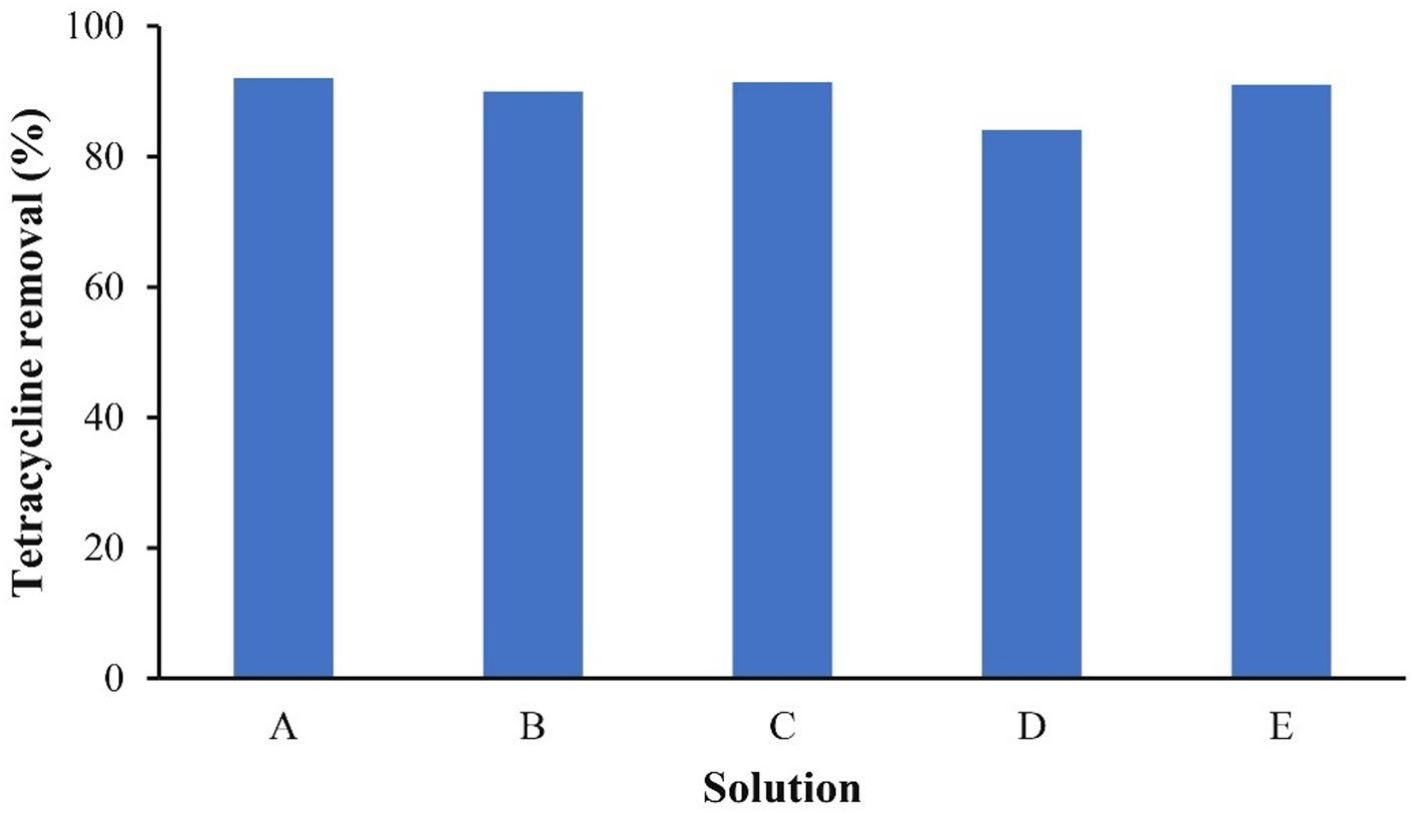
TC removal in the presence of various ions; operating conditions: TC initial concentration 5 mg/L, catalyst dosage 4 g/L, pH 7, time 10 min, and under irradiation of 125 W LED lamp; solutions: (**A**) TC, (**B**) TC, Na^+^, Cl^−^; (**C**) TC, Mg^2+^, NO_3_^−^, (**D**) TC, Na^+^, Cl^−^, Mg^2+^, NO_3_^−^, (**E**) TC, tap water.

To evaluate SnO_2_/RGO performance under realistic conditions, TC photodegradation was studied in the presence of Na^+^, Cl^−^, Mg^2+^, NO_3_^−^ ions, and tap water. Figure 13 summarizes the results. For a 5 mg/L of TC solution containing 5 mg/L of Na^+^ and Cl^−^, TC removal of 90% was obtained. With 5 mg/L of Mg^2+^ and 10 mg/L of NO_3_^−^, TC removal reached 91.47%. In a mixture containing all these ions, 84.01% of TC removal resulted. When tap water was used to prepare the TC solution (5 mg/L), the removal yield was 91.09%. Tap water typically contains Na^+^, K^+^, Fe^3+^, Ca^2+^, Mg^2+^, Cl^−^, SO_4_^2−^, PO_4_^3−^, CO_3_^2−^, and trace disinfection by-products such as trihalomethanes^65,66^. The presence of these species did not significantly impact the removal efficiency, indicating the photocatalyst’s robustness under real water conditions.

Additionally, the SnO_2_/RGO photocatalyst was tested for a mixture solution containing 3 mg/L of TC, 1 mg/L of ciprofloxacin, and 1 mg/L of cefixime. The operating conditions were a catalyst dosage of 4 g/L, pH 7, reaction time 10 min, 125 W LED lamp. The removals were 97% for TC, 97.7% for ciprofloxacin, and 97.6% for cefixime.

### SnO_2_/RGO photocatalytic mechanism

Reduced graphene oxide predominantly consists of sp^2^-hybridized carbon networks with π-π conjugation domains, structural defects, and oxygenated functional groups. Upon light irradiation, electrons in the π systems and defect states become photoexcited. These photoexcited electrons endow pristine RGO with inherent photocatalytic activity^67,34^.

Tin (IV) oxide (SnO_2_) with a wide band gap of 3.6 eV is typically active under ultraviolet irradiation^68^. In contrast, the SnO_2_/RGO exhibits photocatalytic activity under visible light as well. Upon exposure to visible light, RGO acts as an effective photosensitizer. Its photoexcited electrons migrate into the conduction band of SnO_2_, thereby generating additional electron-hole (e^−^/h^+^) pairs^69^. This photosensitization process occurs because the Fermi level of RGO is more negative than the conduction band edge of SnO_2_^70–72^, enabling the transfer of excited electrons from RGO to the SnO_2_ conduction band. Moreover, the high electrical conductivity of RGO facilitates rapid electron transport and suppresses e^−^/h^+^ recombination.

The electrons react with dissolved oxygen to generate superoxide radicals (O_2_^−^·), while the photogenerated holes oxidize water to produce hydroxyl radicals (·OH). These reactive radical species act synergistically to degrade TC molecules in water.

**Fig. 14:**
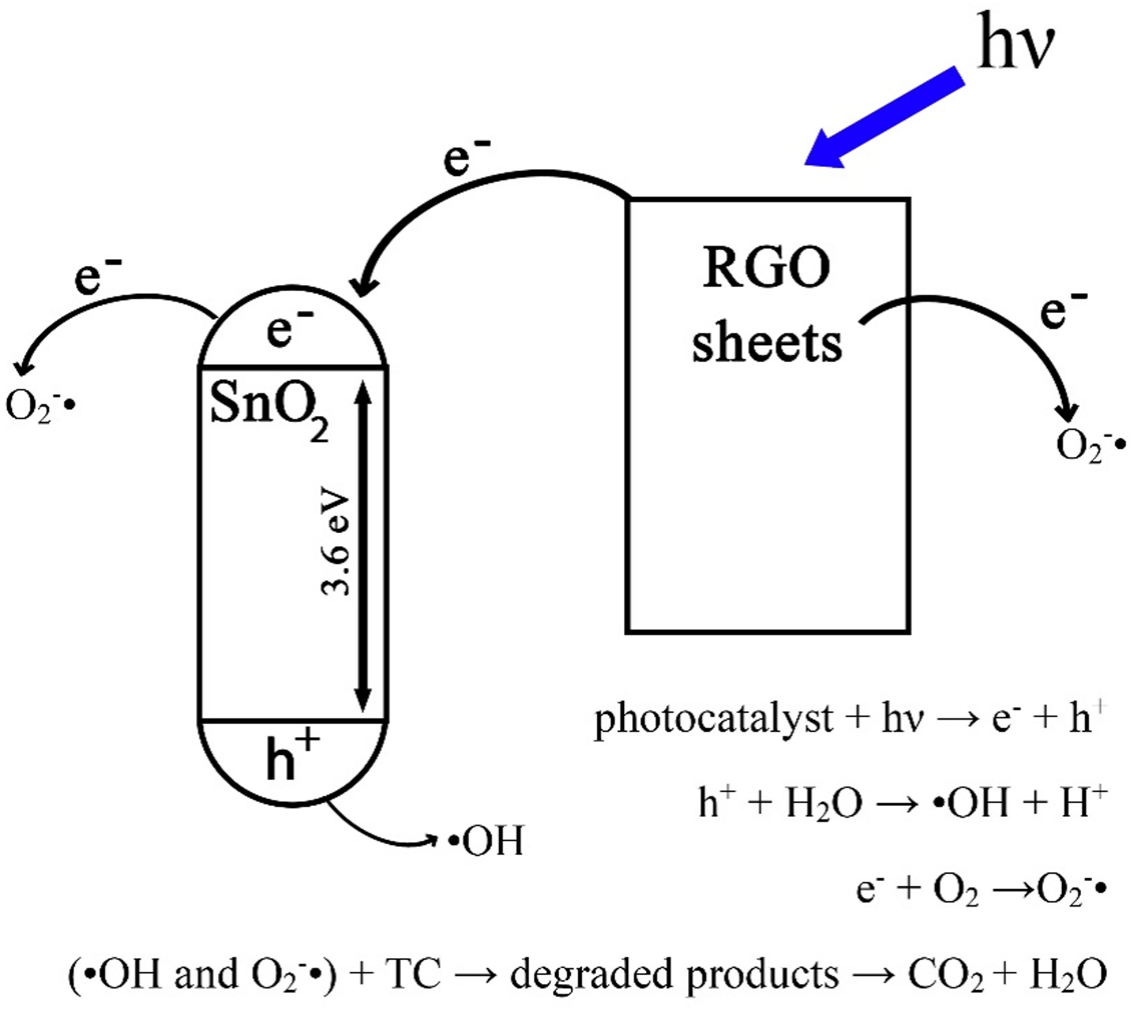
Schematic of the photocatalytic mechanism of SnO_2_/RGO.

The reactions of O_2_^−^· and ·OH radicals with TC lead to bond cleavage, deamination, and ring-opening processes. Further oxidation ultimately results in complete mineralization of TC into carbon dioxide and water^4,23^. Figure 14 shows schematic of the SnO_2_ photocatalytic process.

### Comparison with other studies

The development of photocatalysts that are efficient under visible light, stable, reusable, environmentally benign, and economically viable has been the focus of extensive research. Selected examples from previous studies are summarized in Table 7. The SnO_2_/RGO photocatalyst leveraged the unique properties of RGO, including high electron mobility, large surface area, visible light activity, and excellent structural stability. The photocatalytic degradation kinetics of TC by SnO_2_/RGO were notably fast; in contrast to earlier studies, where the reaction times typically exceeded 60 min. The present photocatalyst, SnO_2_/RGO, achieved comparable removal efficiencies within approximately 10 min. The significant reduction in treatment time allows for reactor size minimization and enhances the overall cost effectiveness of the process. Furthermore, recent advancements in scalable RGO synthesis have made SnO_2_/RGO-based systems more accessible and practical for large-scale water treatment applications.

**Table 7:** Comparison with previous studies.

No.	References	year	Photocatalyst	Operating conditions	TC removal (%)
1	Huang et al.^73^	2022	S-doped BiOBr	PCD^†^: 0.3 g/L, ICTC^‡^: 20 mg/L pH:–, Time: 60 min Xe lamp, 300 W	99.1
2	Jia et al.^74^	2023	Ui66-NDC/P-C_3_N_4_	PCD: 0.05 g/L, ICTC: 30 mg/L, pH:–, Time: 120 min, Xe lamp, 500 W	95
3	Saeunyama et al.^75^	2023	TiO_2_-ZnO	PCD: 0.125 g/L, ICTC: 50 mg/L, pH: 7, Time: 120 min, Xe lamp, 300 W	74
4	Li et al.^76^	2025	Cu_2_O/g-C_3_N_4_	PCD:–, ICTC: 25 mg/L pH:–, Time: 60 min Xe lamp, 100 W	97.5
5	Jiang et al.^77^	2025	Zn/Fe_3_O_4_-modified biochar	PCD: 0.2 g/L, ICTC: 40 mg/L pH:–, Time: 90 min Xe lamp, 300 W	90.4
6	Phakhathi et al.^4^	2025	Porous g-C_3_N_4_ nanosheet	PCD: 1.0 g/L, ICTC: 10 mg/L pH:4, Time: 120 min, Visible lamp, 450 W	83
7	Lu et al.^78^	2025	g-C_3_N_4_/WO_3_	PCD: 1.0 g/L, ICTC: 30 mg/L, pH: 4.4, Time: 180 min, Xe lamp, 300 W	93.3
8	Alotaibi et al.^79^	2025	Iron phosphate	PCD: 0.12 g/L, ICTC: 15 mg/L, pH: 10, Time 300 min, LED lamp, 400 W	87
9	Bahrami et al.	2025	SnO_2_-RGO	PCD: 4 g/L, ICTC: 10 mg/L, pH:7, Time: 10 min, LED Lamp, 120 W	92

^†^Photocatalyst dose, ^‡^Initial concentration of tetracycline.

## Conclusion

Water contamination by antibiotics, particularly TC, contributes to antimicrobial resistance, posing a critical environmental challenge. In this study, SnO_2_/RGO photocatalyst was synthesized via Hummer’s method and optimized for TC photodegradation under visible light. DRS analysis confirmed a reduction in the band gap, enhancing photocatalytic activity compared with pristine RGO and SnO_2_. ICP-OES indicated the SnO_2_ content of 4.34%. XRD analysis confirmed SnO_2_ crystallinity, with no change in SnO_2_ peak intensities and crystallinity size of the used catalyst. SEM and TEM imaging showed uniform dispersion of SnO_2_ nanoparticles over the RGO surface.

Photodegradation kinetics followed a second-order under LED, UV, tungsten, and sunlight irradiation, with LED yielding the highest efficiency. Optimum performance was achieved under 5 mg/L of TC, 4 g/L of photocatalyst, pH 7, and 10 min of LED exposure, with TC removal of 92%. Reusability tests revealed a decline in TC removal from 91 to 57% over five cycles. The photocatalyst maintained its activity in the presence of Na^+^, Cl^−^, Mg^2+^, NO_3_^−^, and tap water. These results demonstrate the potential of SnO_2_/RGO for efficient, rapid, and cost-effective wastewater treatment.

## Supplementary Information

Below is the link to the electronic supplementary material.

Supplementary Material 1

## Data Availability

The datasets generated or analyzed during the current study are available from the corresponding author upon reasonable request.
