# Supplementary material for: Reduced graphene oxide promoted by SnO2 for photodegradation of tetracycline in water
Source: Sci Rep. 2025 Dec 24;16:2511. doi: 10.1038/s41598-025-32230-4 (PMC12820057; doi:10.1038/s41598-025-32230-4)
Supplement: Supplementary file 1 — Supplementary Material 1 [file 41598_2025_32230_MOESM1_ESM.docx]

**Supplementary**

Eq. (S1), COD removal (%) ^63^:

| $COD removal \left( \% \right)=\frac{\mathrm{COD}_{0}-COD}{\mathrm{COD}_{0}}\times100$ | (S1) |
| --- | --- |

Where COD_0_ is the initial chemical oxygen demand and COD is the value after treatment.

Eq. (S2), BOD removal (%) ^64^:

| $BOD removal \left( \% \right)=\frac{\mathrm{BOD}_{0}-BOD}{\mathrm{BOD}_{0}}\times100$ | (S2) |
| --- | --- |

Where BOD_0_ and BOD represent biochemical oxygen demand values before and after photocatalytic degradation.
